# Bone Regeneration Using a Three-Dimensional Hexahedron Channeled BCP Block Combined with Bone Morphogenic Protein-2 in Rat Calvarial Defects

**DOI:** 10.3390/ma12152435

**Published:** 2019-07-31

**Authors:** So-Yeun Kim, Eun-Bin Bae, Jae-Woong Huh, Jong-Ju Ahn, Hyun-Young Bae, Won-Tak Cho, Jung-Bo Huh

**Affiliations:** 1 Department of Prosthodontics, Biomedical Research Institute, Pusan National University Hospital, Busan 49241, Korea; 2 Department of Prosthodontics, Dental Research Institute, Dental and Life Science Institute, BK21 PLUS Project, School of Dentistry, Pusan National University, Yangsan 50612, Korea; 3 Seroun Dental Clinic, Suyeong-ro, Nam-gu, Busan 48445, Korea

**Keywords:** biphasic calcium phosphate, bone regeneration, bone substitute, recombinant human bone morphogenetic protein-2, carboxymethyl cellulose

## Abstract

It is important to obtain sufficient bone mass before implant placement on alveolar bone, and synthetic bone such as biphasic calcium phosphate (BCP) has been studied to secure this. This study used a BCP block bone with a specific structure of the three-dimensional (3D) hexahedron channel and coating with recombinant human bone morphogenetic protein-2 (rhBMP-2) impregnated carboxymethyl cellulose (CMC) was used to examine the enhancement of bone regeneration of this biomaterial in rat calvarial defect. After the preparation of critical-size calvarial defects in fifteen rats, defects were divided into three groups and were implanted with the assigned specimen (n = 5): Boneplant (untreated 3D hexahedron channeled BCP block), Boneplant/CMC (3D hexahedron channeled BCP block coated with CMC), and Boneplant/CMC/BMP (3D hexahedron channeled BCP block coated with CMC containing rhBMP-2). After 4 weeks, the volumetric, histologic, and histometric analyses were conducted to measure the newly formed bone. Histologically, defects in the Boneplant/CMC/BMP group were almost completely filled with new bone compared to the Boneplant and Boneplant/CMC groups. The new bone volume (*P* < 0.05) and area (*P* < 0.001) in the Boneplant/CMC/BMP group (20.12% ± 2.17, 33.79% ± 3.66) were much greater than those in the Boneplant (10.77% ± 4.8, 16.48% ± 9.11) and Boneplant/CMC (10.72% ± 3.29, 16.57% ± 8.94) groups, respectively. In conclusion, the 3D hexahedron channeled BCP block adapted rhBMP-2 with carrier CMC showed high possibility as an effective bone graft material.

## 1. Introduction

In dental clinics, implant procedures have become routine, and most often, and almost uniquely, the most limiting factor is when a large amount of bone is lost. If enough bone is available, the difficulty of the implant procedure will be greatly reduced [1]. In reality, however, the bone volume is often small-scale due to various complications, such as periodontitis, trauma, and alveolar bone resorption due to a long edentulous period. What is required as an ideal bone graft material for this purpose is a scaffold that promotes new bone formation, acts as a pathway for bone-forming material, can be optimally degraded and replaced with new bone, and has sufficient strength and stability [2,3]. Examples of reconstructed alveolar bone include using a bone graft or guided bone regeneration [4], using frozen and radiation sterilized allogenic bone grafting [5], use of graft materials from animals or synthetic production [6], recent use of marine collagen and substitutes [7], and so on. Among them, until now, the most promising method for gaining the alveolar bone has been ideally presented with an autologous bone graft with osteogenic potential. However, there are concerns about the availability and morbidity of the donor, difficulty in shaping, and complexity of the surgery [8,9]. On the other hand, the application of allografts or xenografts is also limited by the drawbacks of immune reaction complications, disease transmission, and the absence of osteogenetic properties that could lead to bone resorption and nonunion [10,11].

New bone graft materials such as 3D scaffolds have been recently studied in the dental field as alternatives to overcome the weak points of previous graft materials. As an alternative to bone grafts of sufficient size and diversiform shape, synthetic bone grafts are becoming increasingly popular because they are capable of sufficient production and have no immune reactions [12,13,14,15]. Block structures instead of powders contribute to stability by themselves. Depending on the size and shape, as well as the architecture and geometry design, they may promote the clustering of stem cells or osteoprogenitor cells and induce them to function as attachment elements within the scaffold [16]. The internal porous structure helps the circulation of tissue ingrowth, body fluids, and nutrients, and the 3D environmental factors, such as pore size and orientation, also influence the tissue regeneration broadly [17]. At this time, aligned pores are more advantageous for cell migration than irregular pores [18].

Meanwhile, biphasic calcium phosphate (BCP), a synthetic bone graft material, is a mixture of stable phase hydroxyapatite (HA) and soluble phase beta tricalcium phosphate (β-TCP) that is widely used because of its biocompatible, osteoconductive, bioactive, safe, and predictable properties [19,20]. BCP ceramic is considered a promising scaffold for use with tissue engineering strategies for large bone augmentation. In dentistry, BCP bioceramic is also recommended as an alternative or supplementary material for autogenous bone [21]. The biomaterial should have a clinically manageable macrostructure, and a microstructure that induces cell adhesion and proliferation [22]. Because their chemical properties, size, and shape can be easily controlled, they have become a multipurpose matrix for bone formation and development [19].

However, to date, the osteoinductivity of biomaterials is still limited and shows a large difference compared to the one with rhBMP-2 [23]. The recombinant human bone morphogenetic protein-2 (rhBMP-2), an osteoinductive growth factor, is sometimes used in the dental field [24,25]. It has a high bone regeneration capability, which differentiates mesenchymal stem cells (MSCs), transforms pre-osteoblasts into osteoblasts, and acts as a trigger for migration of osteoblasts [26,27]. This leads to early stage osteogenic differentiation of stem cells and osteoprogenitor cells to initiate bone formation [28,29]. Meanwhile, the uncontrolled release of rhBMP-2 causes numerous complications, such as heterotopic ossification, osteolysis, or cancer [30,31]. Despite these problems, rhBMP-2 has outstanding effects on bone defect reconstruction and implant osseointegration acceleration, so research is continuing into developing an effective delivery system for its release without adverse effects. [8,32]. A carrier maintains the release at the local site for the time required to induce bone regeneration [8]. A number of delivery systems composed of various materials have been studied for controlled sustained release of rhBMP-2 [33]. Carboxymethyl cellulose (CMC), which is used as a carrier in this study due to its non-toxicity, biodegradability, and hydrophilic characteristics, is a polysaccharide additive used recently in various industries [34,35,36,37,38]. CMC itself has the ability to contribute directly to the synthesis process of calcium phosphate and can be an effective template for biomimetic mineralization. In studies evaluating CMC as a carrier for rhBMP-2, the results showed that alkaline phosphatase activity of fibroblast cells was increased when rhBMP-2 and CMC were used separately, but CMC combined with rhBMP-2 exhibited much better effects [39,40]. By adding CMC to recombinant human osteogenic protein, the quality of union, total histologic appearance, handling characteristics, and stability are reported to be improved in a previous study [41]. According to Yoo et al. [42], in micro-computed tomography and tissue morphometry analysis, BCP with cross-linked CMC promoted new bone formation and increased new bone area ratio. In addition to maintaining the graft volume, CMC has been shown to promote bone metabolism, resulting in the formation of thick new bone around the graft materials in BCP mixed with cross-linked CMC.

The 3D scaffold synthetic block bone can be made with the desired structure and porosity, and can contain the desired biological materials [43]. Pae et al. [44] reported favorable osteogenetic activity of a 3D hexahedron channeled BCP block bone, the same material with used in this study. In comparison with the particle bone, it showed unremarkable new bone formation similar to the experimental group. This block bone is three-dimensionally connected with a tubule with a concave inner surface, made in line with regularity, and has a porosity of 95%. Even when broken, due to the structural characteristics, it was not completely flawed and had the strength to maintain the volume. Three factors affecting the osteoinduction of biomaterials are chemical composition, macrostructure, and surface structure, and this graft material used in this study was expected to meet these requirements [45]. Another previous study that used this 3D hexahedron channeled synthetic bone block obtained favorable but not particularly high amounts of new bone volume in a rabbit calvarial defect model [44]. Therefore, in addition to the volume maintenance ability of this scaffold, we planned to gain more new bone through delivering the CMC and rhBMP-2, and develop a better new bone graft material. Therefore, using this 3D block bone graft material, in this study, we compared the bone formation capacity of in vivo rat calvarial defect model by combining CMC and rhBMP-2 to increase the bone regeneration ability of this 3D hexahedron channeled synthetic BCP block bone.

## 2. Materials and Methods

### 2.1. Preparation of 3D Hexahedron Channeled Synthetic Block Bone

**Figure 1:**
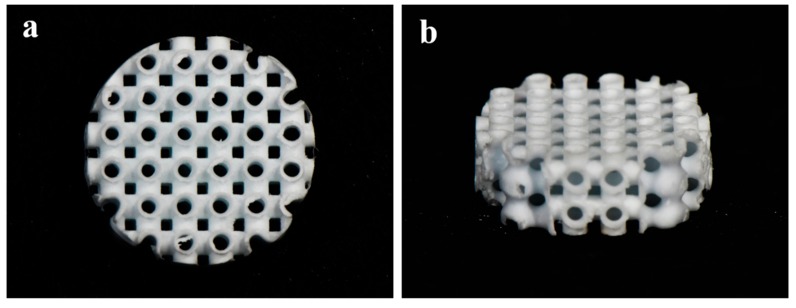
The images of BCP block bone with a 3D hexahedron channel. (**a**) Top view; (**b**) top-side view.

All the specimens of 3D Hexahedron Channeled synthetic block bone were supplied (BoneplantTM, Ezekiel, Chungchung-Namdo, Korea) after sterilization using gamma irradiation. The 3D hexahedron channeled bone block was made of BCP microporous ceramics, with a mixture of 60% HA and 40% β-TCP. The pore size of the disc-shaped bone block (diameter: 8 mm, thickness: 2 mm) was 400–700 μm with 95% porosity (Figure 1). Prior to the animal experiment, the bone blocks were coated with 100 μL of CMC (1.5%, CowellMedi, Busan, Korea) or CMC containing 5 μg of rhBMP-2 (CowellMedi, Busan, Korea) in a completely sterile laboratory [39,46]. The low-level sonication was performed to uniformly coat CMC on the Boneplant surface. The experimental groups of this study were as below:

- Boneplant group: Untreated 3D hexahedron channeled BCP block;
- Boneplant/CMC group: 3D Hexahedron channeled BCP block coated with CMC;
- Boneplant/CMC/BMP group: 3D Hexahedron channeled BCP block coated with CMC containing rhBMP-2.

### 2.2. Scanning Electron Microscope Surface Analysis

The specimens were observed using a Field Emission Scanning Electron Microscope (FE-SEM, S-4700, Hitachi, Tokyo, Japan) to assess morphologies of surface microstructures at magnifications of ×40, ×250, and ×1000. The samples of each group were coated with platinum in ion sputter (E1010, HITACHI, Tokyo, Japan) and were observed using FE-SEM at accelerating voltage of 5 kV.

### 2.3. In Vivo Animal Study

#### 2.3.1. Experimental Animals

Fifteen 12-week-old male Sprague-Dawley rats (5 per group) (Koatech, Pyeongtaek, Korea) weighing 250–300 g were used for this animal experiment. The animals adapted for at least 7 days before surgery and were caged individually with rodent pellets and water supplied ad-libitum. Animal management and surgical procedures were performed at the Pusan National University Laboratory Animal Resource Center and all the experiments followed routines approved by the Institutional Animal Care and Use Committee of Pusan National University (PNU-2018-2101).

#### 2.3.2. Surgical Procedures

**Figure 2:**
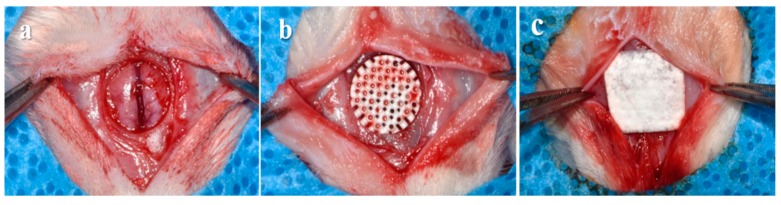
Surgical procedures of this animal experiment. (**a**) Bone defect formation on the cranium. (**b**) Implantation of randomized block bone. (**c**) Placement of collagen membrane.

All the rats were anesthetized by general intramuscular injection of a mixture (0.1 mL/10 g) of Tiletamine-zolazepam (Zoletil50, Virbac Korea, Seoul, Korea) and xylazine (Rompun lnj, Bayer Korea, Seoul, Korea) during the surgical procedures (Figure 2). The surgical sites were clearly shaved and scrubbed with Betadine^®^ (povidone-iodine) for disinfection. The local anesthesia was applied using lidocaine (2% Lidocaine HCl and Epinephrine Injection (1:100,000), Yuhan, Seoul, Korea). After making a sagittal incision across the middle of the cranium, the full-thickness flap was elevated to expose the calvarial bone. A critical-sized circular bony defect (8 mm in diameter) was created in the middle of the cranium using a saline-cooled trephine bur (Osung, Kimpo, Korea). Each defect was randomly implanted with an assigned bone block, and covered with a membrane (10 × 10 mm, collagen membrane, GENOSS, Suwon, Korea). The skin and periosteum were respectively closed using absorbable sutures (4-0, Vicryl^®^, Ethicon, NJ, USA). After 4 weeks, all if the rats were sacrificed by CO_2_ asphyxiation and the surgical sites with surrounding bone were carefully harvested. The obtained tissue samples were placed in 10% neutral-buffered formalin (Sigma Aldrich, St. Louis, MO, USA) for 14 days.

#### 2.3.3. Micro-Computed Tomography (μCT) Analysis

**Figure 3:**
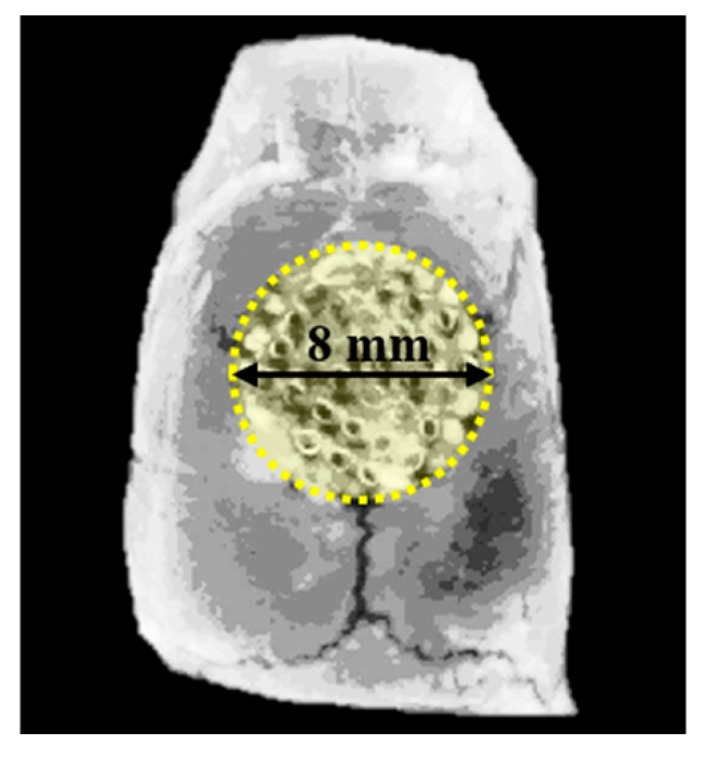
Region of interest (ROI) for micro-computed tomography (μCT) analysis.

To measure the new bone volume within the defect area, the specimens were imaged by micro-computed tomography (SMX-90CT, Shimadzu, Kyoto, Japan) at 90 kV, at an intensity of 109 μA. The region of interest (ROI) was set equal to bony defect size (diameter of 8 mm, height of 1.5 mm) (Figure 3). The images distinguished mineralized bone, soft tissues, and scaffold by adjusting the threshold. The percentages of new bone volume were calculated by customized program corded by cording software (MATLAB 2018a, MathWorks, Natick, MA, USA).

#### 2.3.4. Histologic and Histometric Procedures

**Figure 4:**
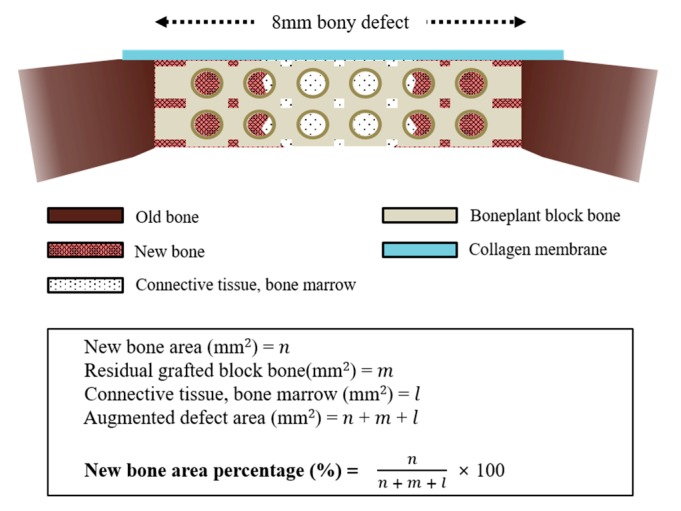
Schematic design for histometric analysis.

The harvested tissue samples were sequentially dehydrated with ethanol (70, 80, 90, and 100%) and infiltrated with embedding methylacrylate-based resin (Technovit 7200 VLC, Heraeus Kulzer, Dormagen, Germany) and then hardened in an ultraviolet polymerization unit (EXAKT 520, Exakt-Apparatebau, Norderstedt, Germany). The final tissue slides with a thickness of 30 μm were prepared from initial sections with 400 μm thickness by cutting and grinding procedures using a microtome (KULZER EXAKT 300, Exakt-Apparatebau, Norderstedt, Germany) and grinding machine (KULZER EXAKT 400CS, Exakt-Apparatebau, Norderstedt, Germany). After staining the specimens with hematoxylin and eosin (H&E) solution, all of the sections were photographed using a microscope (BX51, Olympus, Tokyo, Japan) with a digital CCD camera (Polaroid, MA, USA) For histometic analysis, the newly formed bones within defects were measured using an i-solution image analysis program (IMT, Daejeon, Korea) by a single investigator (Figure 4).

### 2.4. Statistical Analysis

Resulting data of each analysis are expressed as mean ± standard deviation (SD). The in vivo results were analyzed by Kruskal-Wallis test with post-hoc Mann-Whitney test using statistical program (SPSS 25.0, SPSS, IL, USA). *P*-values < 0.05 were considered statistically significant.

## 3. Results

### 3.1. Observations of Surface Morphology

**Figure 5:**
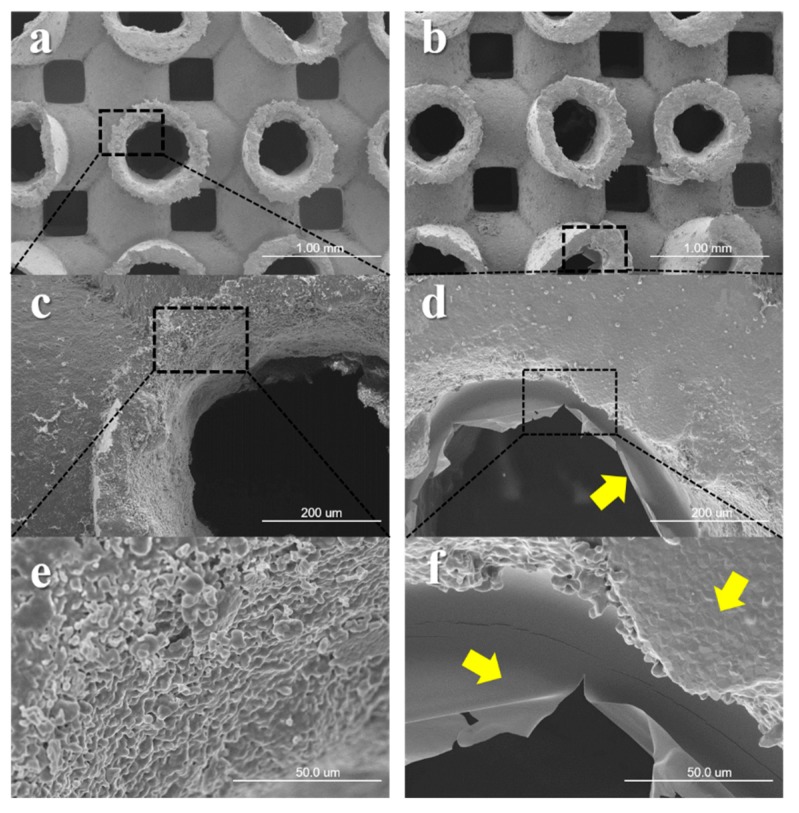
Captured images using scanning electron microscopy. (**a**,**c**,**e**) Boneplant group. (**b**,**d**,**f**) CMC coated groups, Boneplant/CMC, and Boneplant/CMC/BMP group. Yellow arrow: coated CMC on Boneplant surfaces [Original magnifications: ×40 (**a**,**b**) ×250 (**c**,**d**), and ×1000 (**e**,**f**)].

SEM analysis was used to examine the surface morphologies of Boneplant group and CMC coated groups (Boneplant/CMC and Boneplant/CMC/BMP group). Boneplants showed the 3D hexahedron channel (pore size of 400–700 μm; Figure 5a,b). The rough surfaces were observed in the Boneplant group via aggregation of BCP particles (Figure 5c,e). In CMC coated groups, smooth surface were observed covered with CMC compared to the Boneplant group (Figure 5d,f). 

### 3.2. In Vivo Animal Study

#### 3.2.1. Clinical Findings

All of the animals survived during the healing periods without adverse effects, such as infection, inflammation, and specimen exposure at surgical sites.

#### 3.2.2. Volumetric Findings

**Figure 6:**
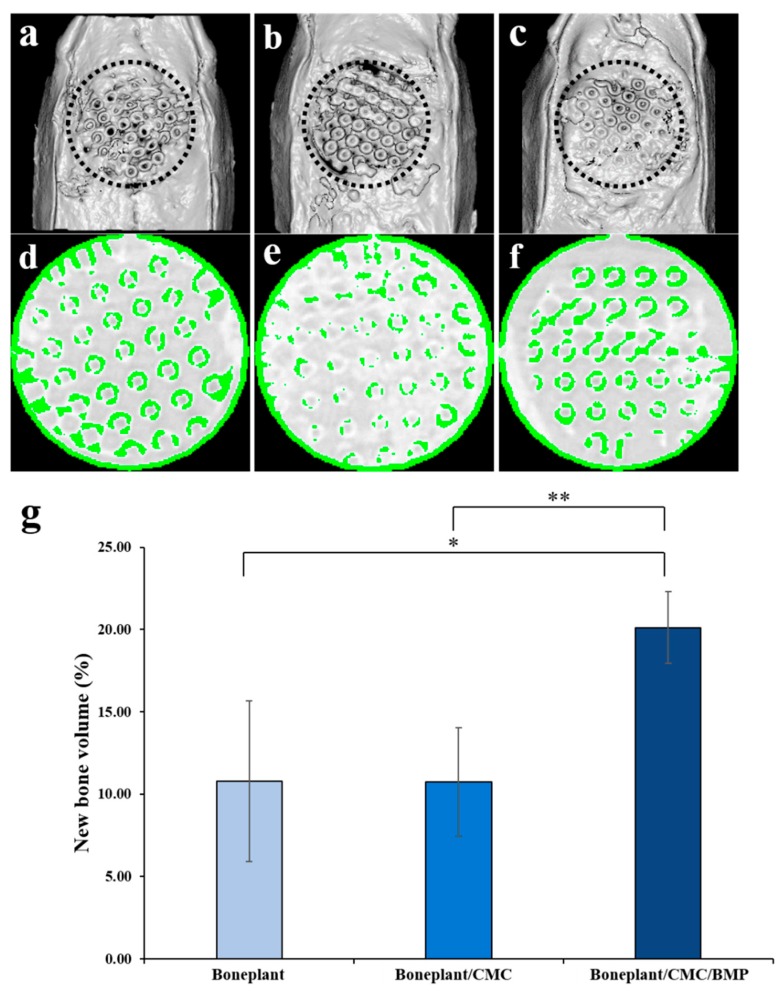
Micro-computed tomography (μCT) findings. (**a**–**f**) Obtained μCT images of each group. (**a**,**d**) Boneplant; (**b**,**e**) Boneplant/CMC; (**c**,**f**) Boneplant/CMC/BMP groups. (**a**–**c**) 3D-reconstructed μCT images. (**d**–**f**) colored μCT images. Green colored area: newly formed bone. Gray colored area: grafted block bone. (**g**) New bone volume within region of interest. The symbol * indicates statistical significance (*P* < 0.05). The symbol ** indicates statistical significance (*P* < 0.01).

The volumetric results of new bone in the Boneplant, Boneplant/CMC, and Boneplant/CMC/BMP groups were 10.77% ± 4.87, 10.72% ± 3.29, and 20.12% ± 2.17, respectively (Table 1, Figure 6g). The newly formed bone was detected between 3D channel pores, especially in the Boneplant/CMC/BMP group (Figure 6a–f). The Boneplant/CMC/BMP groups showed higher new bone volume than the others (*P* < 0.05). However, there was no significant difference between Boneplant and Boneplant/CMC groups (*P* > 0.05).

#### 3.2.3. Histological Findings

**Figure 7:**
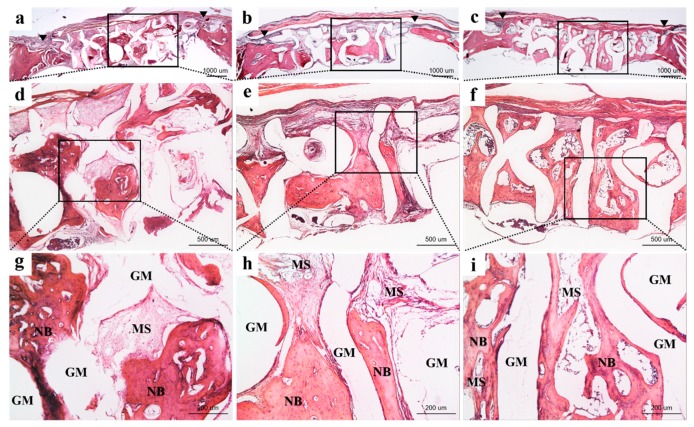
Representative tissue slides at 4-weeks post-surgery: (**a**,**d**,**g**) Boneplant; (**b**,**e**,**h**) Bonplant/CMC; and (**c**,**f**,**i**) Boneplant/CMC/BMP groups. Note: NB = new bone; MS = marrow space; GM = bone graft material. Arrow head = margin of defect (H&E stain, original magnification: (**a**,**b**,**c**) ×12.5, (**d**,**e**,**f**) ×40, (**g**,**h**,**i**) ×100).

The histological abnormal findings such as inflammatory response were not observed in any tissue samples, and implanted bone graft materials were not collapsed and showed good space maintenance in bony defects (Figure 7). In all three groups, the newly regenerated bones infiltrated into the pores of the 3D hexahedron channel structure. In Boneplant and Boneplant/CMC groups, bone marrow and connective tissues were detected around the new bone. The defects of the Boneplant/CMC/BMP group were almost filled with new bone.

#### 3.2.4. Histometric Findings

**Figure 8:**
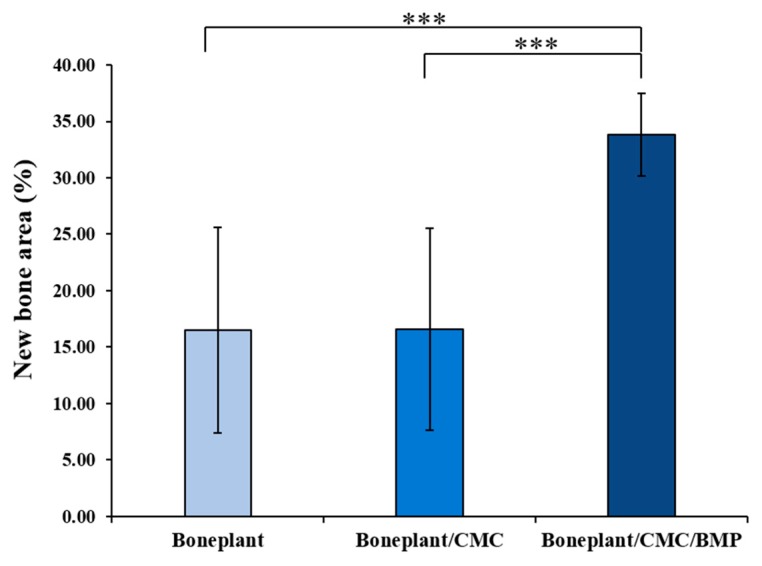
New histometrical bone area percentages of each group. The symbol *** indicates statistical significance (*P* < 0.001).

The histometric results of measuring the new bone area at 4-weeks post-surgery are shown in Table 2 and Figure 8. The Boneplant/CMC/BMP group exhibited more than two-times more new bone area percentage compared with the other groups, indicating statistically different significance (*P* < 0.001).

## 4. Discussion

Bone grafting is a difficult task in dental practice, but it is an essential goal to be solved. Various methods and materials for bone grafting have been studied, but finally in this study the focus is on the use of originated bone graft materials in the laboratory, i.e. synthetic bone [47]. Therefore, in this study, we tried to study more stable and predictive bone graft materials and methods by evaluating new bone formation ability using synthetic bone.

Synthetic bone can be made in the form of a block bone, which is advantageous for volume maintenance, and there is no need to worry about additional surgery or lack of bone quantity. In addition, structural morphology, such as porosity, pore size, interconnectivity, and orientation of synthetic bone, affect bone regeneration [48,49]. According to Frame et al. [50], during the alveolar bone augmentation, grafting of compact solid HA alloplastic bone showed less bone formation than that of porous HA alloplastic bone graft. The scaffold-type synthetic bone produced in 3D can be given a variety of internal structures, such as tube, pore, etc. In the pore structure, fluids and nutrients flow into it and circulate [51]. Micropores permit the capture and concentration of proteins that induce differentiation upon contact with undifferentiated cells [52,53]. The pore enables molecular transport necessary for bone regeneration, and an interconnected internal structure increases cell attachment rate [54]. Studies have shown that compared to the convex surface, the concave surface promotes cell attachment and proliferation and is in charge of the initiation of the bone formation process [52,55]. The pipe-shaped concave surface in the scaffold used in this study is a favorable form for cell attachment and proliferation. The regular hexahedron is connected with empty pipes and the pipe channels are connected in and out. The blood circulates in and out of the six directions of the pipe structure. Bone materials do not roll down because of the matrix structure of the block, which has a sealing effect when it breaks. When it is pushed, highly reactive surfaces are attached to each other to maintain volume.

Micro CT analysis showed no difference between Boneplant and Boneplant/CMC groups, but Boneplant/CMC/BMP group showed differences in new bone volume production from the other two groups. Boneplant alone showed favorable new bone formation when used alone, but rhBMP-2 using CMC as a carrier showed more bone formation. This was also evident in the area value assessed by histometric finding. The difference between the Boneplant and Boneplant/CMC groups was not found, but the new bone area of Boneplant/CMC/BMP group was twice as large as the other two groups. This suggests that a larger area of new bone was obtained when rhBMP-2 was used, which is consistent with the volume results from the micro CT analysis. This volumetric analysis has the limitation that it could not distinguish the tissues perfectly, but it is considered that a relative comparison could be conducted because we used a single set of equipment and the same analysis level for each tissue in order to distinguish the tissues as clearly as possible.

The use of Boneplant in histological findings was found to be superior to the maintenance of space and volume at defect sites. There was hardly any depression on the superior or inferior sides of the defect site. In all three groups, new bone appeared inside the pore, but Boneplant/CMC/BMP groups were filled with new bone, while Boneplant and Boneplant/CMC groups were found to have more connective tissue around the new bone. The pore size of the scaffolds has been shown to affect nutrient transport during tissue regeneration, as well as cell adhesion and migration [56,57]. The appropriate pore size for bone tissue engineering varies slightly depending on conditions, such as 200–400 μm [14], interconnected pore of 300–500 μm [48], 100–300 μm, 600 μm [58], and aligned channel of 270 μm [18], and a clear consensus has yet to be reached. However, if the pore is too large, the blood flow is excessive and it is difficult for the cell differentiation to mature, resulting in a lot of fibrous tissue. On the other hand, if the pore is too small, the pore is clogged before the bone regeneration is completed [18]. The pore size of the block bone used in our study ranged from 400 to 700 μm. Since the channel is not one-way but is six-directional, it is expected that the blood flow was not excessively fast, but it is estimated that some connective tissues were formed inside the pore because of its large pore size. In the Boneplant/CMC/BMP group, rapid differentiation of osteogenic cells was induced by rhBMP-2, suggesting less formation of connective tissue and better quality of direct contact in the pores of new bone.

Among the various biomaterials, the ceramics, especially calcium phosphate, have the advantages of mechanical strength, high affinity for proteins, lower degradation rate, and biocompatibility, so this is the most interesting bone substitute nowadays [59]. Kruyt et al. [60] compared the BCP, β-TCP, and HA with the bone growth ability, and the result showed higher bone growth in BCP and β-TCP. Lim et al. [61] compared the loading of rhBMP-2 or platelet-rich plasma (PRP) in the β-TCP scaffold and the bone regeneration effect of rhBMP-2 was much higher. Magne et al. [62] reported that the BCP loaded directly with human growth hormone showed non-constant hormone release, which was rapid in the first 48 hours and slowed down after this. Calcium phosphate can be 3D printed to have pores and this porosity can be a stimulant of osteogenetic behavior. In the previous study, osteogenic and volume abilities were favorable for bone regeneration in BCP block bone having a 3D hexahedron channel structure [44]. In this study, CMC and rhBMP-2 were loaded for faster bone regeneration, and bone maturity and bone mass were significantly increased when rhBMP-2 was added. Releasing a substantial portion of rhBMP-2 and discharging it in a sustained release pattern is helpful for osseointegration and regeneration of the bone defect around the implant [63]. Herford et al. [32] evaluated the effect of rfBMP-2 in the distraction osteogenesis procedure. They concluded that the addition of rhBMP-2 in the distraction osteogenesis technique induces rapid bone regeneration and soft tissue healing in the bone defect site. On the other hand, locally high concentrations due to short half time induce osteoclasts [64]. Therefore, it is important to maintain sustained release at a constant rate using the carrier for release [65]. The effects of diverse delivery systems on growth factors have been studied. In particular, hydrogel [66], collagen sponge [67], and an altered scaffold surface were presented to prolong growth factor release while avoiding primary massive release [68]. In this study, CMC was applied as a carrier for the sustained release. The addition of rhBMP-2 with CMC resulted in a better quality of new bone distribution with less formation of connective tissue in the graft site.

As a limit in this study, firstly, in the animal experiment, the number of the experimental group is the minimum number, and long-term observation could not be performed because it takes time to use large animals and wait to sacrifice them. In animal studies, systematization of overall animal testing, including appropriate drug use and sacrifice timing, customized for each clinical setting will further improve the predictability of animal clinical trials. In addition, rhBMP-2 showed the potential of added autoinducing in this study and proved its potential, but no resolution of carcinogenesis, dosage, long-term results, and suitable carriers has been reported. We did not investigate the kinetic release of the rhBMP-2 in this study and instead referred to the dose concentration used in the previous article [69]. It would be meaningful to measure the releasing profile of rhBMP-2 in the Boneplant/CMC/BMP group, but this study excluded this test because the difference between fast release and slow release of rhBMP-2 was not the main evaluation variable. Eventually, this is the fundamental problem of exposing too much rhBMP-2 to the local area, and additional and continuing research on the appropriate dose and delivery system of rhBMP-2 to biomaterial is needed.

## 5. Conclusions

Bone formation was assessed by grafting using a 3D BCP synthetic block bone with an internal hexahedron channel structure. When the 3D hexahedron channeled BCP block bone was implanted, the volume of the bone was secured to 50% or more of the transplantation site, and BCP itself was effective as a transplanted bone at the defect site to maintain volume and produce new bone. The addition of rhBMP-2 with CMC showed significant volume and a large area of new bone. The addition of rhBMP-2 reduced the formation of connective tissue inside the pores and improved the quality of the new bone. Therefore, 3D hexahedron channeled BCP block combined with rhBMP-2 is expected to have a better effect for bone regeneration during bone grafting, and further studies are required for other types of 3D BCP block bone with rhBMP-2.

## Figures and Tables

**Table 1:** New bone volume within regions of interest (n = 5 per group).

	Groups	Mean	SD	*P*-Value
**New Bone Volume (%)**	Boneplant	10.77	4.87	0.013 *
	Boneplant/CMC	10.72	3.29	
	Boneplant/CMC/BMP	20.12	2.17	

The symbol * indicates statistical significance (*P* < 0.05).

**Table 2:** New bone area percentages within the region of interest (n = 5 per group).

	Groups	Mean	SD	*P*-Value
**New Bone Area (%)**	Boneplant	16.48	9.11	0.000 ***
	Boneplant/CMC	16.57	8.94	
	Boneplant/CMC/BMP	33.79	3.66	

The symbol *** indicates statistical significance (*P* < 0.001).
